# Perspective: the top 11 priorities to improve trauma outcomes, from system to patient level

**DOI:** 10.1186/s13054-022-04243-2

**Published:** 2022-12-21

**Authors:** Michael C. Reade

**Affiliations:** 1 Medical School, University of Queensland, Level 9 Health Sciences Building, Royal Brisbane and Women’s Hospital, Herston, QLD 4029 Australia; 2 Joint Health Command, Australian Defence Force, Canberra, ACT 2610 Australia

**Keywords:** Wounds and injuries, Trauma, Emergency medical care, Military medicine, Resuscitation, Advanced trauma life support care, Quality improvement, Clinical trial, Accident prevention

## Abstract

**Background:**

The Haemorrhage, Airway, Breathing, Circulation, Disability, Exposure/Environmental control approach to individual patient management in trauma is well established and embedded in numerous training courses worldwide. Further improvements in trauma outcomes are likely to result from a combination of system-level interventions in prevention and quality improvement, and from a sophisticated approach to clinical innovation.

**Top eleven trauma priorities:**

Based on a narrative review of remaining preventable mortality and morbidity in trauma, the top eleven priorities for those working throughout the spectrum of trauma care, from policy-makers to clinicians, should be: (1) investment in effective trauma prevention (likely to be the most cost-effective intervention); (2) prioritisation of resources, quality improvement and innovation in prehospital care (where the most preventable mortality remains); (3) building a high-performance trauma team; (4) applying evidence-based clinical interventions that stop bleeding, open & protect the airway, and optimise breathing most effectively; (5) maintaining enough circulating blood volume and ensuring adequate cardiac function; (6) recognising the role of the intensive care unit in modern damage control surgery; (7) prioritising good intensive care unit intercurrent care, especially prophylaxis for thromboembolic disease; (8) conducting a thorough tertiary survey, noting that on average the intensive care unit is where approximately 15% of injuries are detected; (9) facilitating early extubation; (10) investing in formal quantitative and qualitative quality assurance and improvement; and (11) improving clinical trial design.

**Conclusion:**

Dramatic reductions in population trauma mortality and injury case fatality rate over recent decades have demonstrated the value of a comprehensive approach to trauma quality and process improvement. Continued attention to these principles, targeting areas with highest remaining preventable mortality while also prioritising functional outcomes, should remain the focus of both clinician and policy-makers.

## Background

At the level of an individual patient, protocols for investigating and treating patients after severe physical trauma are beguilingly straightforward: stop major haemorrhage, open the airway while protecting the cervical spine, ensure adequate breathing, maintain an adequate circulation, assess disability and act to reduce further neurological damage, and expose the patient to identify all injuries while paying attention to environmental temperature so as not to worsen hypothermia. These concepts have now been taught to thousands of clinicians worldwide in Advanced Trauma Life Support courses [1] and their derivatives. Such priorities arose in an era when it was sufficient for medical practice to “make sense”, without the need for comparative evidence generated from randomised controlled trials. Perhaps this is appropriate when logic and benefit are so clear. However, clinical innovations in modern trauma care—such as retrograde endovascular balloon occlusion of the aorta, or various prehospital transfusion interventions—are expensive and seek progressively smaller incremental improvements in outcome. Accordingly, many believe these require evaluation in randomised controlled trials with cost-effectiveness analyses. However, trauma presents challenges to conventional clinical trial design: patient heterogeneity, the need to randomise within a short time window, inability to obtain patient (and often substitute decision-maker) consent, the typically brief period of the experimental intervention, and outcomes affected by multiple factors other than the intervention all work together to make it difficult to observe the effect of any experimental treatment. With notable exceptions, most clinical trials in trauma have not shown a benefit of what appeared promising interventions. Despite this, population mortality and case fatality rate from accidental injury are falling dramatically in many trauma systems worldwide. If individual clinical interventions are not responsible—what is? This paper explores the answer to this question, seeking evidence for what might be considered the top 11 priorities for trauma systems worldwide.

## Invest in trauma prevention (1)

**Fig. 1:**
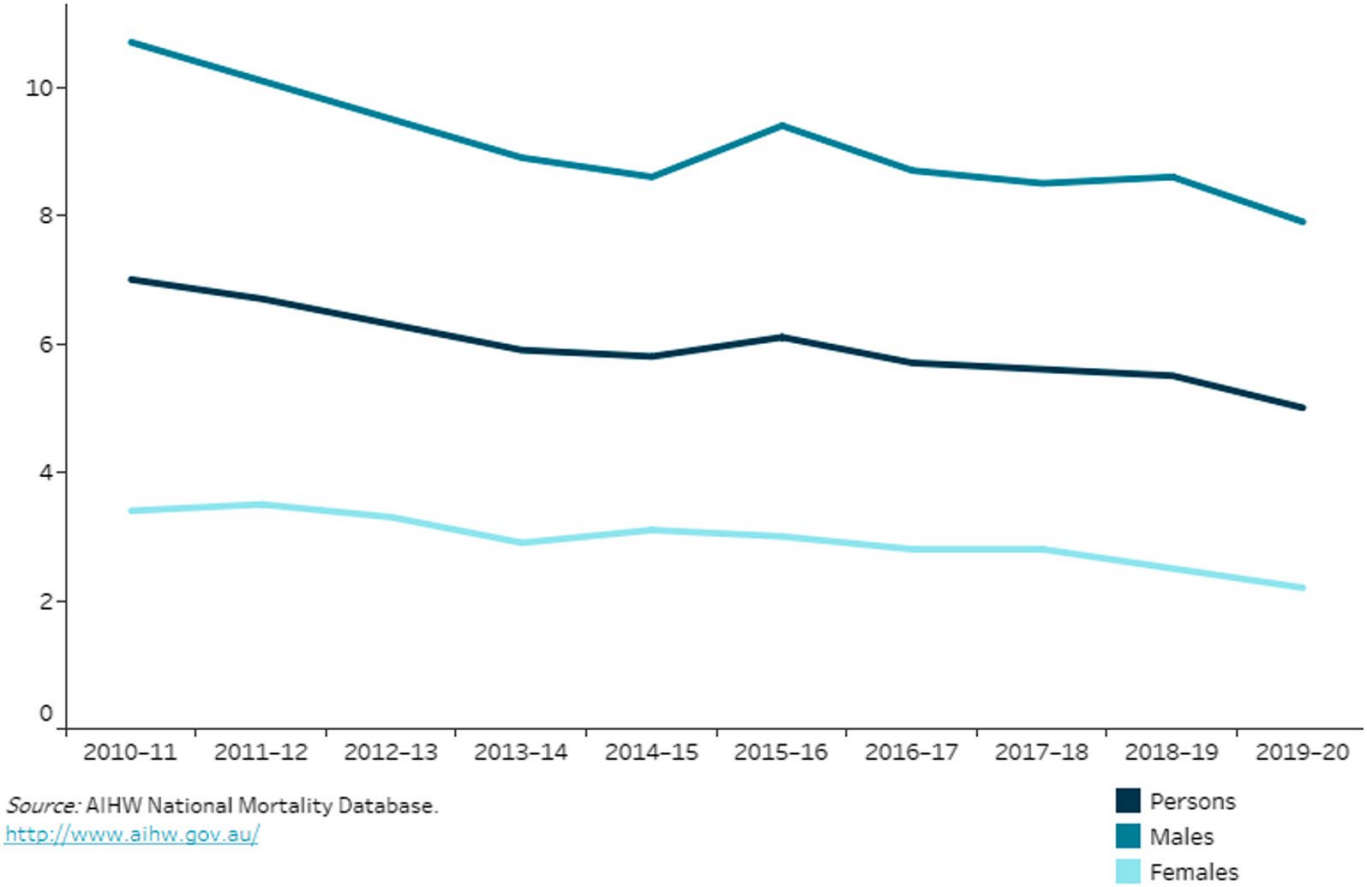
Transport injury hospitalisations and deaths, by sex, 2010–11 to 2019–20 [2]. Reproduced under a Creative Commons BY 4.0 license

Injury constitutes 8.4% of the burden of disease in Australia, responsible for 2,100 hospitalisations per 100,000 population in 2019–2020. The 13,400 deaths made “injury” (the definition of which includes suicide and accidental poisoning) the leading cause of death for people aged 1–44 years, a population rate essentially unchanged over the preceding 10 years [2]. Attempts to improve this statistic must look at specific causes, as interventions to reduce intentional self-harm in young adults will differ from programs to reduce road traffic crashes or falls in the elderly. Public health interventions can succeed: when transport injury (the commonest cause of accidental injury in younger people) is examined in isolation, the average annual reduction in Australian population mortality between 2010–11 and 2019–20 was -3.7% (Fig. 1) [2]. US Department of Transport data is not so encouraging, showing an *increase* in deaths per 100 million miles travelled from 1.10 in 2011 to 1.37 in 2020 [3]. Various reasons for Australia’s success have been suggested, including enforcement of laws regarding alcohol, drugs, and mobile telephones while driving, seat-belt and helmet laws, and investment in road infrastructure [4]. The science of injury prevention fills textbooks; suffice to note the conclusion that in trauma “the most effective medical and cost reduction strategy would be prevention” [5]. Clinicians have a vital role: while epidemiologists and experts in public policy are essential, bringing statistics to life with individual patient stories is critical in affecting policy and behaviour change.

## Invest in prehospital care (2)

**Fig. 2:**
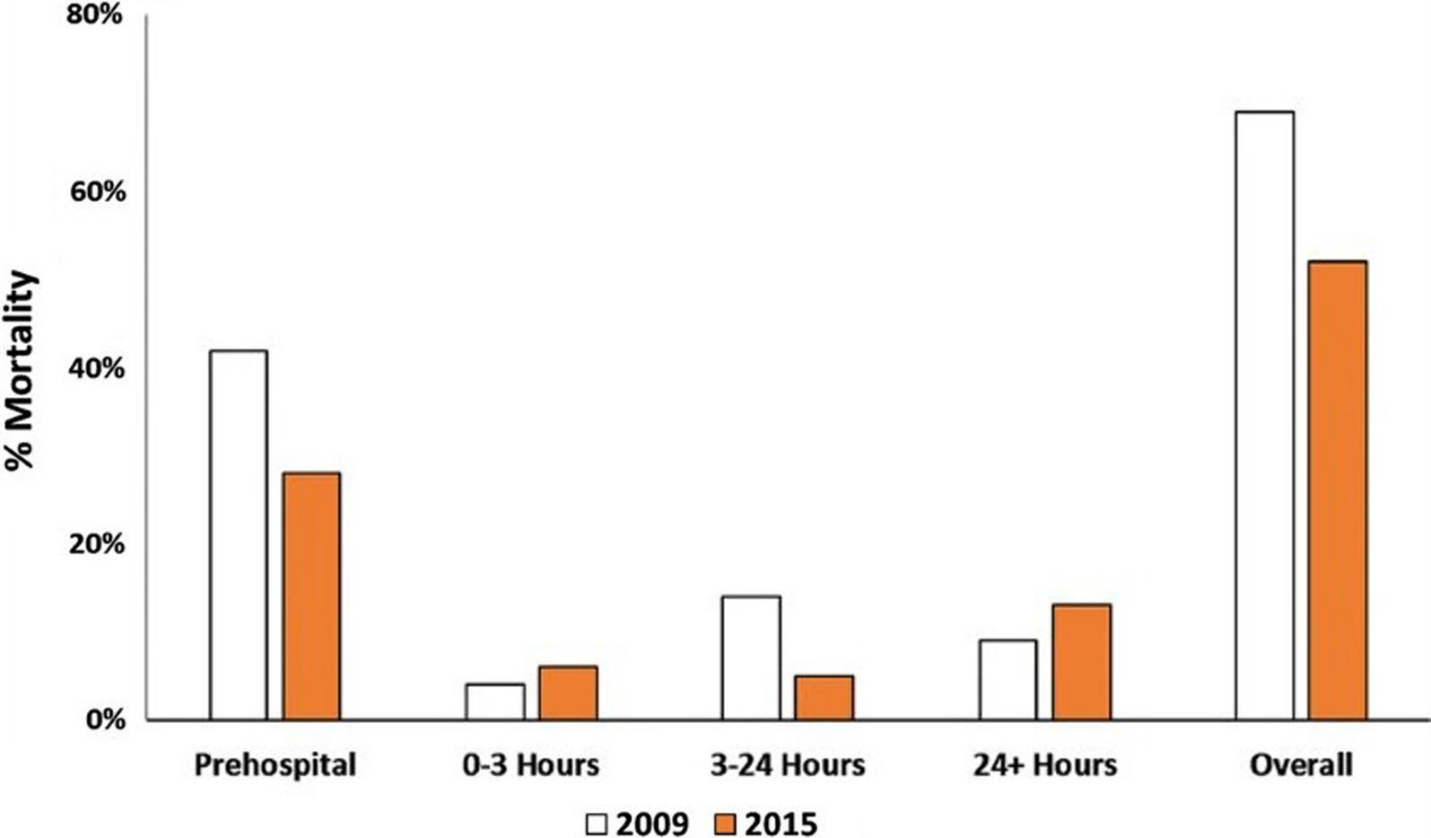
Time distribution of mortality of patients activating a major haemorrhage protocol and receiving at least one unit of red blood cell transfusion. Overall mortality reduced 25% from 2009 to 2015. A drop in prehospital mortality resulted in a 50% higher death rate immediately post-admission. Improvements in management of coagulopathy dramatically reduced mortality rates between 3 and 24 h but were associated with a 44% increase in later deaths [10]. Image Copyright © 2019, Springer-Verlag GmbH Germany, part of Springer Nature

In addition to improvements in population mortality, introduction of comprehensive trauma systems is associated with improvement in case fatality rate. Severity- and age-adjusted odds of death fell by approx. 50% in Victoria, Australia between 2001–2 and 2008–9 [6], although this dramatic improvement has subsequently plateaued. A similar improvement was seen in Israel [7]. In low- and middle-income countries, resources required to produce dramatic improvements are not large. For example, training of villagers accompanied by the introduction of a trauma-focussed protocol for prehospital paramedics saw civilian trauma mortality fall in Northern Iraq 1997–2006 from 17 to 4% [8]. Highly sophisticated military trauma systems can also improve. When a US National Guard unit of civilian-trained Critical Care Flight Paramedics replaced conventional US Army medics on evacuation helicopters, the severity-adjusted odds of 48-h death fell to one-third [9]. Reduction in prehospital deaths can lead to a higher mortality at 24 h, as was seen in London 2009–2015 (Fig. 2), but overall mortality remained lower [10]. Importantly, this London study emphasised that most trauma deaths still occur prehospital, so logically that is where the greatest proportion of preventable deaths remain and consequently where clinical innovation and quality improvement are likely to have the greatest impact. Further, new strategies are required for the sicker patients who are now surviving until later in their hospital course.

## Build a high‑performance trauma team (3)

Teamwork in medicine is sometimes compared to a rowing crew, with each member working in harmony, pulling in the same direction to a common goal. A better analogy might be to a jigsaw puzzle, with pieces of different shapes required to fit together forming a coherent whole. Trauma team members have complementary, not identical, skills. Each team member must have mutual trust and respect, humility and willingness to learn, and be able to communicate effectively. Leading a trauma team involves eliciting the best from every team member, rarely providing technical directions alone. Frequently the team leader is not the most important technical determinant of a patient’s outcome—this might be the surgeon stopping haemorrhage, the anaesthetist securing a threatened difficult airway, or the nurse who recognises an incipient error, or who elicits an essential item of history from the patient. Part of teamwork is delegating tasks to competent team members who work autonomously, only reporting difficulties to the team leader. For example, in many protocols the airway specialist is trusted to open the airway, if possible take a brief history, and intubate when appropriate. The medication doctor or nurse might be authorised by protocol to insert an intravenous cannula and administer drugs that are given to all trauma patients: for example in penetrating trauma a protocolised dose of antibiotic, ketamine, tetanus toxoid and fluid. Such protocols can be practiced in drills with specific designated roles, such as those outlined for the trauma team in the UK military [11].

## Clinical interventions: stop bleeding, open & protect the airway, optimise breathing (4)

While systems are important, so are clinical interventions. Demonstrating effectiveness in trauma clinical trials is challenging for the reasons explored elsewhere in this article, but many fairly recent innovations have entered practice based on preclinical studies, observational evidence, or compelling logic. For example, amongst wounded soldiers in Iraq, those who had windlass arterial limb tourniquets applied prehospital had a higher 25-day survival than those whose tourniquet was applied in hospital [12], with no evidence of increase in limb loss from ischaemia [13]. However, successful placement by untrained civilians is low (11.3–23.5% depending on tourniquet type) [14], suggesting the need for training. Junctional tourniquets occlude arterial vessels at the junction of the trunk and limbs, but are difficult to maintain in place during transport [15]. Pro-coagulant haemostatic dressings are in widespread use by military forces for penetrating trauma [16], with some marginally superior to others in preclinical models. Professional societies [17] have recognised that airway management in trauma requires a different protocol to generic anaesthetic airway management, as patients cannot usually be woken if the airway is unable to be secured (as in elective surgery), cardiovascular instability precludes the use of certain medications, and hypoxia at the time of induction is more common. Consequently, prioritising succinylcholine [18, 19] over non-depolarising muscle relaxants unless contraindicated, removing the cervical collar (if one was applied) and providing manual in-line cervical stabilisation while attempting intubation, routinely using high-flow nasal oxygen and video laryngoscopy during intubation, and quickly excluding a pneumothorax after intubation using ultrasound are all useful recommendations. The greater haemodynamic stability and absence of adrenal suppression associated with ketamine have led many to advocate for its use in trauma rapid sequence induction in preference to propofol or etomidate, although superiority in blood pressure effects [20] or 30-day mortality [21] have not been demonstrated in large observational trauma studies.

## Maintain enough circulating blood volume; ensure adequate cardiac function (5)

The 1994 randomised trial [22] of 598 adults with penetrating torso injuries and a prehospital systolic blood pressure of ≤ 90 mmHg that found a lower hospital mortality (30% vs. 38%, *p* = 0.04) associated with no fluid administration prior to the operating theatre (compared to standard practice, which resulted in a mean of 1608 ml preoperative fluid) argued against the practice of aggressive crystalloid resuscitation. However, patients in the delayed resuscitation group in this trial took a mean of only 27 min to reach the hospital. Logically, there must be a time delay after which at least some preoperative fluid becomes beneficial. This has only been shown in an animal model: pigs exposed to blast/haemorrhage then 60 min of hypotensive resuscitation had a substantially lower mortality if then fluid resuscitated to a near-normal blood pressure than those allowed to remain hypotensive [23]. This “hybrid resuscitation” approach should ideally be titrated to markers of organ perfusion in an individual patient.

Choice of resuscitation fluid is the source of considerable controversy. Modern protocols minimise crystalloid in favour of blood components, in part to avoid dilution, but also to preserve or restore the endothelial glycocalyx, which preclinical experiments have shown is not protected by crystalloid resuscitation [24]. Having understood that plasma [25] and platelets [26] are essential components in trauma resuscitation, the choice is between a ratio-guided approach, viscoelastic-guidance, or unfractionated whole blood. The ITACTIC trial [27] was not able to show superiority of a viscoelastic-guided approach in major haemorrhage, which might be related to the difficulties of trials in general. Similarly, meta-analyses have found no consistent evidence supporting the superiority of whole blood over component therapy [28, 29]. The ideal ratio of red cells to plasma and platelets remains unclear after the 680-patient PROPPR trial [30] did not find clear superiority of a 1:1:1 over a 1:1:2 plasma/platelet/red cell protocol.

If wishing to minimise crystalloid resuscitation, it is useful to be able to predict which patients will ultimately require transfusion of substantial quantities of blood products based on data available at the time of their admission to hospital. Two scoring systems facilitate this: the simpler Assessment of Blood Consumption (ABC) Score [31] (Table 1) and the Trauma Associated Severe Haemorrhage (TASH) Score [32].

**Table 1:** The assessment of blood consumption (ABC) score [31]

SBP ≤ 90 mmHg	+ 1
HR ≥ 120 bpm	+ 1
Positive FAST scan	+ 1
Penetrating torso injury	+ 1

Total score	Incidence of major transfusion
0	2%
1	10%
2	38%
3	42%
4	100%

While focussed on circulation, it is important to remember the possibility of myocardial dysfunction and injury. Small myocardial tears were present in 0.3–0.9% of major blunt chest trauma patients [33]. A normal ECG and clinical signs did not exclude pericardial tamponade, which should therefore be suspected in all fluid non-responders. Most (70–80%) patients survived with thoracotomy or pericardiotomy and oversew of the tear. Echocardiography can also identify hypovolaemia and inadequate contractility (possibly as a consequence of contusion) and so should be a mandated component of the extended Focussed Assessment by Sonography in Trauma (eFAST) examination.

## Recognise the role of the ICU in modern damage control surgery (6)

The term “damage control” was first applied in the surgical literature in 1992 to a case series of 46 patients [34], although the concept of abbreviated surgery aiming only for haemostasis and control of contamination followed by a period of physiological stabilisation had been advocated some years earlier. While that original manuscript made no military allusions, the naval concept of damage control (which in order prioritises “float, move, and fight”) is analogous to the surgical aims of preserving life, minimising further damage, and restoring function. Understanding, and so preparing for, the role of the ICU in this process is essential. For example, the UK military reported on experience managing 43 casualties with bilateral limb amputations from blast. Their median ICU stay was 7 days, with a median of 8 operations undertaken in a staged damage control then reconstructive approach [35].

The goal of early ICU care is sometimes said to be to reverse the “lethal triad” of hypothermia, acidemia and coagulopathy caused by dilution of clotting factors. While each of these features is certainly associated with poor outcomes (e.g. in a study of 604 trauma patients who underwent massive transfusion, hypothermia of < 34 °C was associated with lower survival [36]), it does not necessarily follow that these associations are causative. The clinical importance of the lethal triad is to identify the most unwell patients, not necessarily to serve as independent therapeutic targets. A more sophisticated understanding of the pathophysiology involved is the key to better treatments.

Several experiments illustrate this point. Ex vivo analysis of human blood found clotting time was not affected until temperature fell substantially below 34 °C. At 33 °C, the rate of thrombin generation was near-identical to that at 37 °C [37]. Furthermore, it does not necessarily follow that rewarming would reverse whatever pathophysiological process had been established by cold. Similarly, experimentally induced ex vivo acidemia paradoxically increased, rather than decreased, the rate of thrombin generation, potentially due to a decrease in antithrombin activity [38]. Clinicians understand that artificial correction of pH in an acidemic trauma patient using bicarbonate does not correct coagulopathy. The 1198-patient PROMMTT observational study found that coagulopathic trauma patients did have slightly lower clotting factor levels compared to trauma patients who were not coagulopathic, but probably to a degree insufficient to explain their coagulopathy [39]. Fibrinogen, often said to be the factor that falls first in trauma coagulopathy, was paradoxically increased. The explanation for coagulopathy in this study was a dramatic elevation in activated protein C, from a mean 8.1 to 37.2 units (*p* < 0.001).

Activated protein C is central to the theory of Acute Traumatic Coagulopathy [40], in which factor Va and VIIIa are deactivated and the inhibition of plasminogen activator inhibitor is removed, activating plasmin to cause fibrinolysis. Understanding this mechanism suggests therapeutic targets likely to be more effective than rewarming, factor replacement and bicarbonate. Instead, the endothelial glycocalyx must be preserved to prevent the exposure of thrombomodulin from activating protein C. This is best achieved by maintaining perfusion (and so maintaining normothermia, and preserving an adequate circulating blood volume with plasma or whole blood rather than crystalloid) and inhibiting plasmin activation by administering tranexamic acid as early as possible [41, 42].

## Prioritise good ICU intercurrent care, especially venous thromboembolism (VTE) prophylaxis (7)

Other texts provide more detail on the benefits of good general intensive care in trauma; suffice to note that as in other aspects of ICU practice, a systematic approach, potentially assisted by checklists and audits, improves outcomes such as infection [43]. One particular element of intercurrent ICU care warrants attention: VTE prophylaxis. Severe trauma patients switch from a coagulopathic to a procoagulant state at an early (but difficult to identify) time in their recovery [44]. The incidence of deep venous thrombosis (DVT) after major trauma is up to 5.1% despite prophylaxis, with studies of higher-risk patients reporting an incidence of up to 44% [44]. VTE has the potential to cause substantial morbidity, and despite treatment pulmonary embolus or in situ pulmonary thrombosis has a case fatality rate in trauma nearing 50% [44]. Pulmonary embolus is the third most common cause of death in trauma patients who survive beyond 24 h [44]. Despite convincing trial evidence in trauma patients from 1996 showing a significantly lower incidence of DVT in patients commenced on low molecular weight heparin (LMWH) (compared to unfractionated low-dose heparin) within 36 h, [45] in many hospitals VTE prophylaxis is delayed or LMWH avoided for fear of bleeding. Consensus guidelines provide specific recommendations for even high-risk patients [46], incorporating evidence from the largest randomised trial of inferior vena cava filters that found no evidence of benefit from early placement in high-risk trauma patients with a contra-indication to anticoagulation [47].

## Conduct a thorough tertiary survey (8)

An essential part of modern ICU management of major trauma is to seek occult injuries missed in the abbreviated assessment performed prior to the life-saving interventions immediately prior to ICU admission. Numerous institutional checklists are available (e.g. [48]) to facilitate thorough assessment, usually accompanied by CT or MRI. A systematic review [49] found nine studies that reported rates of missed injuries later identified in tertiary survey, with a hospital-weighted rate of 4.3% but with rates at individual hospitals ranging from 1.26% to 65%. The average reported rate in included studies was 15.7%. Training interventions reported in three studies showed significant improvements (from 3% identified to 7% identified).

## Facilitate early extubation (9)

Many major trauma patients are young and were previously physically fit, making them resilient to physiological stress. This makes early extubation possible, even if return to surgery for a staged procedure is planned within 24–48 h. However, early extubation requires adequate analgesia (for which regional techniques are often particularly appropriate [50]), adequate chest wall mechanics in the case of rib fractures (suggesting possible benefit of early rib fixation in select patients [51]), and recognition that an abdominal wall with a temporary closure device (such as a Vacuum-assisted closure (VAC) dressing) does not mandate ongoing mechanical ventilation [52]. More than any other factor, the intensive care team need to overcome the natural inertia to leave a patient intubated between operations.

## Invest in quality assurance and improvement (10)

Hospitals that underwent the process of American College of Surgeons trauma centre accreditation reduced their severity-adjusted trauma length of stay by 10% (*p* < 0.000), the ratio of observed/expected mortality from 0.81 to 0.59 (*p* < 0.000), and costs by 5% (*p* < 0.000) [53]. The UK military introduced a comprehensive set of trauma performance process and outcome indicators spanning prehospital to ward care [11], which was associated with a year-on-year progressive increase in the Injury Severity Score at which 50% of severely injured patients died [54]. While these processes were unable to identify exactly which interventions were improving, that realisation itself is instructive. The greatest improvements in trauma outcomes are likely to result from closer adherence to a culture of excellence than to the introduction of any single therapeutic intervention.

## Improve clinical trial design (11)

While this paper has identified numerous examples of how trauma care has been improved in certain circumstances that are likely to translate more broadly, very few randomised controlled trials showing superiority in mortality outcomes underpin this evidence base. Indeed, the only trial showing benefit from any transfusion intervention is the PAMPer study of prehospital plasma, which assessed mortality at 30 days [55]. In-hospital administration of tranexamic acid required a study of 20,000 patients to demonstrate 28-day mortality benefit [42]. Experience has therefore shown that expecting to observe the effect of a brief intervention applied to a heterogenous patient population many days later, through the “noise” of many intercurrent influences on relevant observable non-continuous outcomes such as mortality, is highly ambitious. Such a trial requires either a large treatment effect size or a large sample size. Alternatives are to accept the validity of non-patient-centric surrogate outcomes (such as markers of coagulopathy, or amount of fluid transfused) or, perhaps most controversially, aim only for very short-term benefit such as 6-h all-cause mortality, as has recently been proposed [56]. Whatever the strategy, it should be clear that persisting with traditional trial designs appropriate for other circumstances is unlikely to succeed in trauma.

## Conclusions

While injury case fatality rate is falling in many parts of the world, trauma remains the leading cause of death for younger patients and the cause of substantial lifelong disability for many more. Addressing this complex problem requires an integrated complex approach, involving better population-based prevention strategies, more effective clinical interventions, and system-level improvements in the way trauma care is delivered, particularly prehospital. Eventually, investments will reach a point of diminishing return, but there is little evidence in trauma that this point is yet near.

## Data Availability

Not applicable.

## Abbreviations

CT: Computerised tomography
DVT: Deep venous thrombosis
eFAST: Extended Focussed Assessment by Sonography in Trauma
ICU: Intensive care unit
LMWH: Low molecular weight heparin
MRI: Magnetic resonance imaging
VAC: Vacuum-assisted closure
